# A knowledge translation tool improved osteoporosis disease management in primary care: an interrupted time series analysis

**DOI:** 10.1186/s13012-014-0109-9

**Published:** 2014-09-25

**Authors:** Monika Kastner, Anna M Sawka, Jemila Hamid, Maggie Chen, Kevin Thorpe, Mark Chignell, Joycelyne Ewusie, Christine Marquez, David Newton, Sharon E Straus

**Affiliations:** Institute of Health Policy, Management and Evaluation, University of Toronto, Toronto, Ontario Canada; Li Ka Shing Knowledge Institute of St. Michael’s Hospital, Toronto, ON Canada; Division of Endocrinology, University Health Network and University of Toronto, Toronto, ON Canada; Department of Clinical Epidemiology and Biostatistics, McMaster University, Hamilton, ON Canada; Dalla Lana School of Public Health, University of Toronto, Toronto, ON Canada; Department of Mechanical and Industrial Engineering, University of Toronto, Toronto, ON Canada; Faculty of Medicine, University of Toronto, Toronto, ON Canada

**Keywords:** Knowledge translation, Clinical decision support, Chronic disease management, Primary care, Risk assessment, Osteoporosis, Interrupted time series analysis

## Abstract

**Background:**

Osteoporosis affects over 200 million people worldwide at a high cost to healthcare systems, yet gaps in management still exist. In response, we developed a multi-component osteoporosis knowledge translation (Op-KT) tool involving a patient-initiated risk assessment questionnaire (RAQ), which generates individualized best practice recommendations for physicians and customized education for patients at the point of care. The objective of this study was to evaluate the effectiveness of the Op-KT tool for appropriate disease management by physicians.

**Methods:**

The Op-KT tool was evaluated using an interrupted time series design. This involved multiple assessments of the outcomes 12 months before (baseline) and 12 months after tool implementation (52 data points in total). Inclusion criteria were family physicians and their patients at risk for osteoporosis (women aged ≥50 years, men aged ≥65 years). Primary outcomes were the initiation of appropriate osteoporosis screening and treatment. Analyses included segmented linear regression modeling and analysis of variance.

**Results:**

The Op-KT tool was implemented in three family practices in Ontario, Canada representing 5 family physicians with 2840 age eligible patients (mean age 67 years; 76% women). Time series regression models showed an overall increase from baseline in the initiation of screening (3.4%; P < 0.001), any osteoporosis medications (0.5%; P = 0.006), and calcium or vitamin D (1.2%; P = 0.001). Improvements were also observed at site level for all the three sites considered, but these results varied across the sites. Of 351 patients who completed the RAQ unprompted (mean age 64 years, 77% women), the mean time for completing the RAQ was 3.43 minutes, and 56% had any disease management addressed by their physician. Study limitations included the inherent susceptibility of our design compared with a randomized trial.

**Conclusions:**

The multicomponent Op-KT tool significantly increased osteoporosis investigations in three family practices, and highlights its potential to facilitate patient self-management. Next steps include wider implementation and evaluation of the tool in primary care.

**Electronic supplementary material:**

The online version of this article (doi:10.1186/s13012-014-0109-9) contains supplementary material, which is available to authorized users.

## Background

Over 200 million people worldwide have osteoporosis, representing a considerable health care and financial burden [1–6]. The aging of the population will likely further compound the disease burden [2,3,7]. Fragility fractures are the clinical consequence of osteoporosis, with vertebral and hip fractures having the most devastating prognosis [8] and being associated with an increased risk of death [9]. Such fractures can significantly impair quality of life, physical function, and social interaction and can lead to admission to long-term care [10–12]. Clinical practice guidelines are available for osteoporosis management [13–15], but their implementation in clinical practice has been inconsistent, such that many patients are still not receiving appropriate diagnostic testing or treatment [16–20]. Less than 40% of patients receive appropriate therapy [17]; and the proportion of patients with fragility fractures who receive a diagnostic test or a diagnosis from a physician is not optimal (range 1.7% – 50%) [18–20]. Knowledge translation (KT) tools such as clinical decision support systems may be one solution to closing these practice gaps because they can provide evidence at the point of care to facilitate disease management. Clinical decision support systems generate patient-specific assessments or recommendations for clinicians by means of software algorithms that match information from a knowledge database to relevant clinical data and provide evidence-based suggestions for assessment and treatment [21–23].

In response to existing management gaps, we developed an osteoporosis KT (Op-KT) tool using two theoretical frameworks: the knowledge-to-action framework of Graham et al [24] and the Medical Research Council (MRC) framework for complex interventions [25]. The knowledge-to-action framework consists of knowledge creation (which allows knowledge to be filtered down from inquiry and synthesis into tools that can aid in decision making) and a series of iterative and dynamic action steps to apply this knowledge [24]. Importantly, the framework highlights the need to involve relevant end-users of the knowledge that is being implemented [24]. The MRC framework was also considered because it complements the iterative, phased approach of the knowledge-to-action framework, and it has the potential to strengthen the development and implementation of KT interventions by addressing challenges to achieving optimal study design, execution, and generalizability [25]. Our knowledge creation process involved conducting a systematic review of randomized controlled trials to determine what features of osteoporosis tools support clinical decision making [26]. We found that few osteoporosis tools exist, but interventions consisting of reminders and education targeted to both physicians and patients had potential for increasing osteoporosis investigations and treatment [26]. We combined these findings with available national osteoporosis guidelines and input from clinicians and experts in information technology and human-factors engineering to design a conceptual Op-KT tool. We then assessed the barriers and facilitators to using this knowledge in a qualitative study of focus groups with family physician [27], which enabled the development of a prototype. Finally, we tested the prototype in a mixed-methods usability study to ensure that it met the needs of target end-users including patients at risk for osteoporosis and physicians who provide care for them [28]. A description of the Op-KT tool is in the Methods below.

The objectives of the current study were to test the implementability of the multicomponent Op-KT tool and to determine its effectiveness on appropriate management of osteoporosis by family physicians (*i.e.*, initiation of investigations such as bone mineral density testing (BMD) and treatment with medications such as bisphosphonates and nutritional supplements such as calcium and vitamin D).

## Methods

The tool was implemented between May and November 2009 (phase one) and evaluated between July 2009 and November 2010 (phase two). Details of the protocol have been published previously [29]. The study was approved by the University of Toronto Health Sciences Research Ethics Board.

### Phase one: implementation of the Op-KT tool

**Figure 1:**
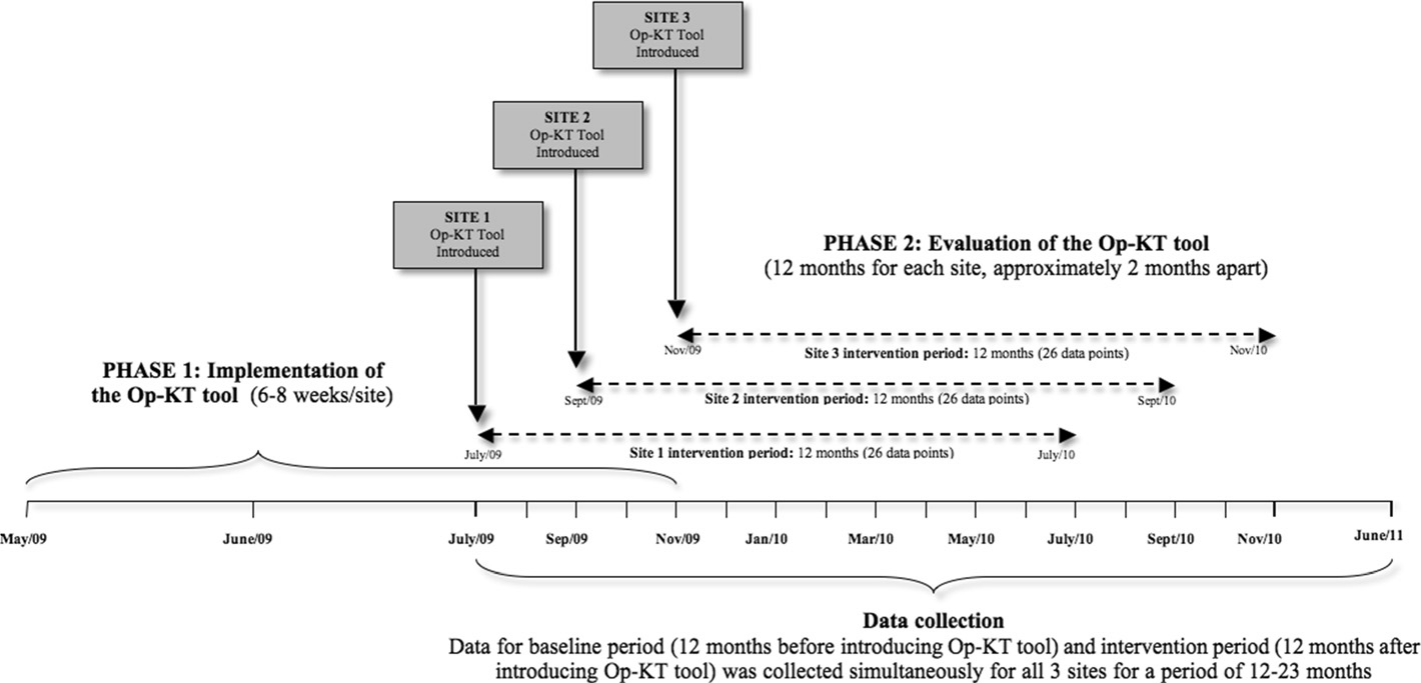
**Study design and flow.**

Our previous work revealed that, for complex interventions intended for delivery at the point of care, workflow must be investigated before implementation [27,28]. As such, we developed an a priori plan involving workflow analysis to determine the feasibility of physicians and patients using the tool at the point of care and to identify factors that would minimize any disruption to usual care caused by the tool. Our goal was to tailor the tool to the practice and workflow of each setting and thereby to facilitate its adoption and uptake during phase two. Implementation involved observation of clinic staff during the patient registration process and estimations of the time patients spent waiting for their physician visit and the length of visits; an environmental scan to ensure appropriate installation of equipment; development of a customized procedures manual with input from clinic personnel; and provision of training to physicians, nurses, and other clinic staff directly involved in use of the tool. We staggered the schedule for implementing the Op-KT tool for each of the three sites (approximately two months apart) to accommodate for the time needed to complete the implementation plan described above (see Figure 1 for study design and flow). The tool was designed to be pragmatic (*i.e.*, there was no deliberate procedure to prompt patients to initiate the RAQ) because workflow analyses indicated a need for seamless integration of the tool. As a result, there were some differences between sites in how the RAQ was initiated and the outputs delivered at the point of care. For example, the two solo practice sites had touchscreen devices in the waiting areas (as well as the examination rooms) to maximize opportunities for patients to use the tool.

### Description of the Op-KT tool

The functional Op-KT tool (accessible at http://knowledgetranslation.ca/osteo_final/) has 3 components:

1. A short (13-item) RAQ that is completed on a touch-screen device by at-risk patients in the waiting room or the examination room before their family physician visit. The RAQ was designed to assess major and minor osteoporosis risks as outlined in the Osteoporosis Clinical Practice Guidelines [30]. Screenshots of the RAQ can be viewed in Additional file 1.
2. A paper-based best practice recommendation prompt (BestPROMPT), generated according to patients’ RAQ responses and outlining appropriate, individualized, guideline-based [30] osteoporosis management recommendations for family physicians at the point of care. An example of this is shown in Additional file 2.
3. A paper-based customized osteoporosis educational sheet (COPE) given to patients at the end of the visit, outlining their individual identified risks and suggestions for managing those risks. An example of this is shown in Additional file 3.

### Setting and population

Three family practices were purposively selected from the Hamilton Family Health Team, which is the largest of the 150 approved primary care Family Health Teams in Ontario, Canada, serving approximately 280 000 people [31]. It includes a comprehensive team of family physicians, nurses, registered dieticians, mental health counsellors, psychiatrists, and pharmacists. We selected 3 family practices in total, as this was sufficient to evaluate the Op-KT tool under different practice settings (one group and two solo) for a pilot study. Those who agreed to participate and met the eligibility criteria were included. Participants were family physicians in solo or group practice using the PracticeSolutions electronic medical record (EMR) system (TELUS Health, Canada) and their patients at risk for osteoporosis (women aged ≥50 years, men aged ≥65 years). A written consent was obtained from all participating physicians.

### Phase two: evaluation of the Op-KT tool

#### Study design and sampling

We used the quasi-experimental design with interrupted time series (ITS) analysis to estimate the effect of the Op-KT tool on appropriate disease management outcomes. The study design and its execution were guided by the ITS quality criteria as outlined by Ramsey *et al.* [32] and the Cochrane Effective Practice and Organisation of Care group [33]. In ITS studies, sample size calculations are related to the estimation of the number of observations or time points at which data will be collected. The literature is variable on the number of data points to consider in different situations, but it is generally recommended that at least 20 pre-intervention and 20 post-intervention observations be used to ensure sufficient power to detect a change and to account for threats to validity, such as trends, seasonal or cyclical observations over time, or random fluctuations with no discernible patterns [32,33]. We used a relatively large number of data points: 26 data points over the 12 months before starting the intervention (baseline period) and 26 data points over the 12 months after implementation (intervention period), for a total of 52 data points for each site (see Figure 1). To increase validity, data points were set closer together (*i.e.*, each data point represented a two-week period rather than four weeks) [34].

### Outcomes

The primary outcomes were initiation of appropriate osteoporosis investigations (*i.e.*, BMD testing) and treatment (*e.g.*, any bisphosphonate or nutritional supplements such as calcium and vitamin D) by family physicians. ‘Appropriate’ osteoporosis management was defined according to clinical practice guidelines available at the start of phase two [30]. Screening and treatment recommendations were used to develop a disease management algorithm [29], which was programmed into the RAQ. Secondary outcomes were the number of patients who completed the RAQ (measured by patient-initiated RAQ logs generated by the touch-screen tablets), the mean time for completion of the RAQ (minutes), and occurrence of fractures. We also assessed osteoporosis risk factors by sex. Using queries in the EMR system and an automated computer program within the touch-screen devices, we also documented any actions related to the primary outcome taken by family physicians of patients who completed the RAQ and collected site-specific demographic data (number of patients in practice, number of age-eligible patients and their mean age, and patients who had at least one visit during the intervention period).

### Unit of analysis and data collection

Our unit of analysis for our primary outcomes was based on the multiple baseline assessment of individual family practice sites. These outcomes were first summarized as rates at each time point, using proportion of osteoporosis investigation and prescriptions, where the denominator represented the number of all eligible visits. We established a stable baseline of standard practice for each site using a pre-intervention chart review. The specific procedure for data collection was published in the protocol [29]. Briefly, two investigators (MK, CM) used patient charts and touch-screen tablet logs to collect visit-specific data bimonthly. Every time a patient completed the RAQ, summaries from physician and patient outputs (BestPROMPT and COPE sheets, respectively) were saved in a folder on the encrypted device hard drive. These summaries captured the date and time the RAQ was initiated and completed and the timed logs of all RAQ responses. We confirmed RAQ use and documented any outcome-related actions taken by physicians using chart review. Our unit of analysis for our secondary outcomes was at the level of patients.

### Statistical analysis

We provided site level as well as overall summary measures, where data was combined across the three sites. For normally distributed continuous data, we reported means and standard deviations and performed t-tests and analysis of variance (ANOVA) to compare differences in means between groups. For non-normally distributed continuous data, we reported medians and interquartile ranges and used nonparametric tests for group comparisons. For categorical data, we reported frequencies and percentages and used Fisher exact tests and chi-square tests for comparing differences between groups.

We used segmented linear regression modeling to examine the effect of the intervention on the primary outcomes [35]. This was done at the site level and overall, where rates of osteoporosis investigations and prescriptions were compared before and after the intervention was introduced; and combined for the overall estimate by averaging across all sites. Three primary outcomes considered were: rates of BMD testing, prescription any osteoporosis medications, and any nutritional supplements. For each outcome, the initial segmented regression model used can be described as:

$$ \mathrm{Y}={\upbeta}_0+{\upbeta}_{1*}\mathrm{time}+{\upbeta}_{2*}\mathrm{Intervention}+{\upbeta}_{3*}\mathrm{time}*\mathrm{Intervention}+\upvarepsilon, $$

Where, Intervention is a dichotomous variable indicating pre- and post-intervention time periods. The coefficients β_0_ and β_1_ represent the constant term (intercept) and trend over time (slope) during the baseline (pre-intervention) period, respectively; whereas, β_2_ and β_3_ represent changes in the level and trend of outcomes after the intervention (compared to baseline level and trend).

We used the Durbin-Watson’s test [36] to examine presence of serial correlation in the residuals after fitting the segmented regression model. When statistically significant autocorrelation is detected, autoregressive integrated moving average (ARIMA) models was used as required (by including a lag term in the regression models model, eg. Y_t_ –Y_t-1_, for first order autocorrelation) using the Cochrane-Orcutt methodology [37]. All statistical analyses were conducted using SAS version 9.3, and statistical significance was set at *P* < 0.05.

## Results

Five family physicians among two solo practices (site one and site two) and one group practice (site three) participated in the study. Table 1 shows the patient population and the number of site visits in the study. Across the three sites, a total of 9,138 and 9,171 patients visited their physician during the pre- and post-intervention periods, respectively. Of these, 2390 patients (81% women) were deemed age eligible (*i.e.*, women ≥50; men ≥65 years of age) during the pre-intervention period; 2,840 patients (76% women) were age-eligible during the post-intervention period (Table 1).

**Table 1:** **Characteristics of the study population across the 3 family health team study sites during the baseline and intervention periods**

**Characteristics**	**Baseline period**	**Intervention period**
Total number of patients in practice	9138	9171
Total number of age-eligible patients* (%)	2,390 (26)	2,840 (31)
Women	1,947 (81)	2,164 (76)
Men	566 (24)	676 (31)
Mean age of patients	67	67
Women	65	65
Men	74	74
Total number of visits (%)	16 283	16 549
Total number of visits by age-eligible patients^†^ (%)	6,139 (38)	6,306 (38)

*Women ≥50 years of age; men ≥65 years of age.

^†^Some patients had more than one visit.

### Primary outcomes—results of the ITS analysis

**Figure 2:**
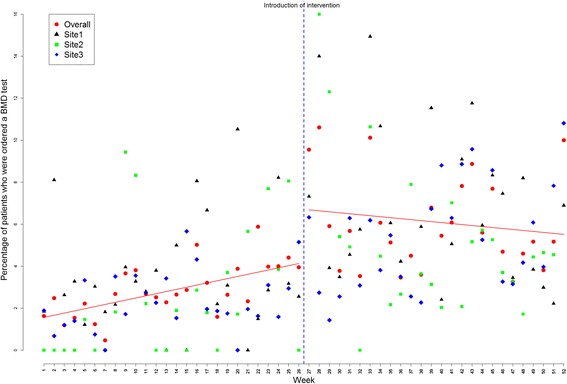
**Percentage of patients for whom a bone mineral density test was ordered: represented for each site as well as for sites overall (July 2009 to November 2010).** The 52 data points represent 26 two-week segments before and 26 two-week segments after introduction of the intervention (vertical rule).

**Figure 3:**
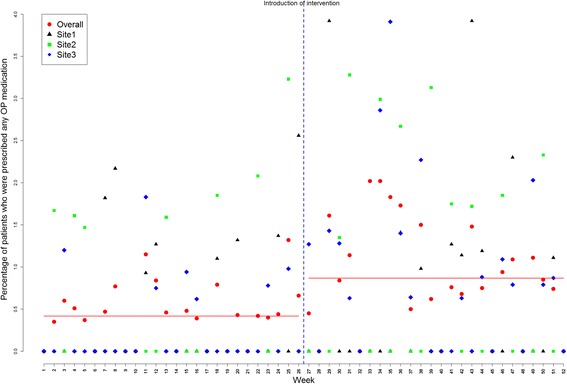
**Percentage of patients for whom any osteoporosis medication was prescribed (bisphosphonates [alendronate, etidronate, risedronate, or zoledronic acid], selective estrogen receptor modulators [** ***e.g.*** **, raloxifene], nasal calcitonin, parathyroid hormone, or hormone replacement therapy): represented for each site as well as for sites overall (July 2009 to November 2010).** The 52 data points represent 26 two-week segments before and 26 two-week segments after the introduction of the intervention (vertical rule).

**Figure 4:**
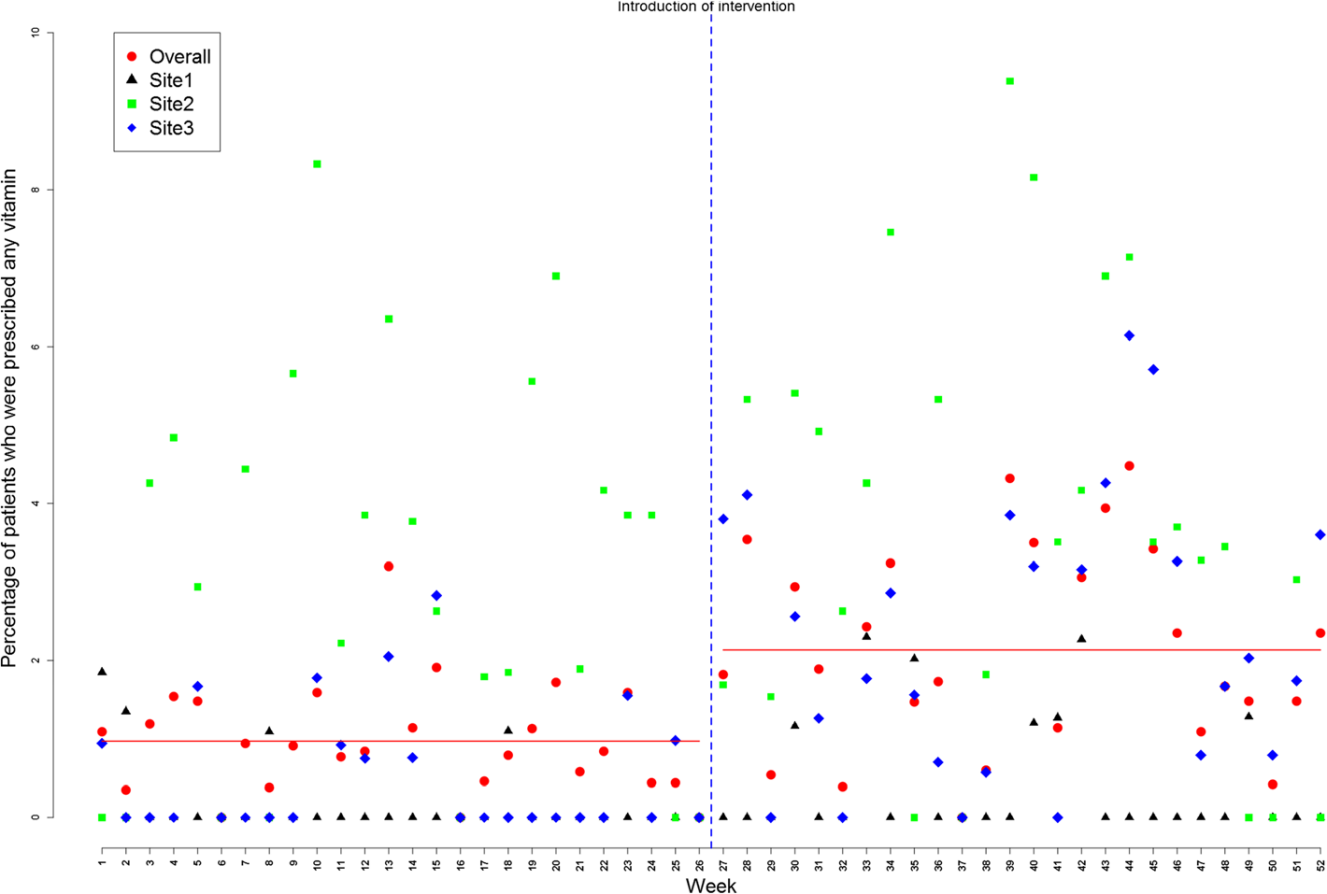
**Percentage of patients for whom any nutritional supplement (** ***i.e.*** **, calcium or vitamin D) was prescribed: represented for each site as well as for sites overall (July 2009 to November 2010).** The 52 data points represent 26 two-week segments before and 26 two-week segments after the introduction of the intervention (vertical rule).

Time series regression models showed an increase in the initiation of BMD testing from baseline of 2.79% during pre-intervention time to 6.15% in post-intervention period (difference of 3.4%, 95% confidence interval [CI 2.03-4.68; p = <0.001), indicating an increase of 34 BMD tests for every 1,000 eligible visits across the three sites. Initiation of any osteoporosis medication increased by 5 in 1,000 eligible visits (difference of 0.5%, CI 0.15-0.85; p = 0.006), and initiation of any nutritional supplement (calcium or vitamin D) by 12 in 1000 eligible visits (difference of 1.2%; CI 0.49-1.91; p = 0.001) (Table 2). Plots of average rates (averaged over the three sites) for the three outcomes are shown in Figures 2, 3 and 4, respectively.

**Table 2:** Results of the interrupted time series analysis for initiation of osteoporosis investigations and treatments, combined across the three sites*

**Outcomes**	**Mean of proportions across the three sites (SD)**		**% Increase**	***P*** **value** ^**†**^
	**Baseline**	**Intervention**		
**Initiation of BMD testing**	**2.79 (1.27)**	**6.15 (2.24)**	**3.4**	<0.001
95% CI	1.86 to 3.74	3.38 to 8.42	2.03 to 4.68	
**Initiation of any osteoporosis medication** ^**‡**^	**0.42 (0.36)**	**0.87 (0.66)**	**0.5**	0.006
95% CI	0 to 1.13	0 to 2.16	0.15 to 0.85	
**Initiation of calcium + vitamin D**	**0.70 (0.56)**	**1.58 (0.95)**	**0.9**	<0.001
95% CI	0 to 1.80	0 to 3.44	0.39 to 1.41	
**Initiation of any nutritional supplement (calcium or vitamin D)**	**0.97 (0.71)**	**2.13 (1.28)**	**1.2**	0.001
95% CI	0 to 2.36	0 to 4.64	0.49 to 1.91	

*BMD, bone mineral density; SD, standard deviation.

^†^From autoregressive integrated moving-average (ARIMA) model. Intercept only model is fitted since the slopes are not significantly different from zero.

^‡^The following osteoporosis medications were considered: any bisphosphonate (alendronate, etidronate, risedronate, zoledronic acid), any selective estrogen receptor modulator (*e.g.*, raloxifene), nasal calcitonin, parathyroid hormone, and hormone replacement therapy.

A statistically significant autocorrelation of order 7 (correlation value of 0.51, 0.22, 0.34 for BMD testing, any osteoporosis medication and nutritional supplement with p-values of <0.001, 0.035, 0.004; respectively) was observed in the residuals; results adjusted for autocorrelation. Further investigation of the residuals (after fitting the adjusted model) showed randomly distributed residuals indicating no residual serial correlation left between the measurements across time.

### Site-level analysis

Site level interrupted time series analysis indicated that the intervention led to improvements in outcomes across all sites, but not all increases were significant. For example, a statistically significant improvement was observed for BMD testing (compared to pre-intervention) in Site 1 (4.29%, CI 1.72-6.86; p = 0.001) and Site 3 (3.07%, CI 1.55-4.59; p <0.001) but not in Site 2 (3.40%, CI -0.43-7.23; p = 0.082). Increases in osteoporosis medication prescriptions in the post- *0intervention period was also significant in Site 3 (0.78%, CI 0.18-1.38; p = 0.011) but not in Site 1 (0.18%, CI -0.35-0.71; p = 0.5023) or Site 2 (0.29%, CI -0.28-0.86, p = 0.3324). Similarly, the prescription of nutritional supplements increased across all sites during the post-intervention period, but this increase was only significant in site three (1.85%, CI 0.86-2.84; p <0.001). All site level analyses were adjusted for autocorrelation, when significant autocorrelations were detected.

The breakdown of the number of eligible visits as well as the number (and rate) of osteoporosis investigations and prescriptions by site and combined sites are provided in Table 3. The results indicate that the median numbers of visits during the pre- and post-intervention times were similar for all three sites. Overall, the median and rate of BMD testing, initiation of osteoporosis medications and supplements were improved during the post-intervention period compared to the pre-intervention period, and these findings are consistent across all (Table 3). For example, the overall rate BMD testing increased during post-intervention compared to the baseline period by 2.9%, suggesting that the intervention led to about 29 more BMD tests for every 1000 eligible visits. The largest increase was observed in Site 3 (*i.e.*, the group practice) where an increase of 31 BMD tests for every 1,000 eligible visits were observed. Relatively smaller improvements (but consistent across all the three sites) were observed for the initiation of osteoporosis medications and nutritional supplements (Table 3).

**Table 3:** Number of eligible visits, and number of visits leading to a primary outcome (BMD testing, initiation of osteoporosis medications and nutritional supplements) across the three sites*

**Site**	**Eligible visits**		**BMD testing**			**Initiation of any osteoporosis medication**			**Initiation of any nutritional supplements (calcium, vitamin D)**		
	**Median (IQR)**	**Total**	**Median (IQR)**	**Total**	**Rate** ^**‡**^ **(95% CI)**	**Median (IQR)**	**Total**	**Rate (95% CI)**	**Median (IQR)**	**Total**	**Rate (95% CI)**
**Combined** ^**†**^											
Overall	240.0 (51.75)	12,445	10.00 (7.00)	550	4.42 (4.07-4.80)	1.00 (2.00)	82	0.66 (0.53-0.82)	3.00 (3.75)	191	1.53 (1.32-1.77)
Pre-Intervention	238.0 (49.75)	6,139	7.00 (5.25)	180	2.93 (2.53-3.39)	1.00 (1.00)	25	0.41 (0.27-0.61)	2.00 (2.25)	60	0.98 (0.75-1.27)
Post-Intervention	247.0 (60.50)	6,306	14.00 (16.25)	370	5.87 (5.31-6.48)	2.00 (2.25)	57	0.90 (0.69-1.17)	4.50 (4.25)	131	2.08 (1.75-2.47)
**Site One**											
Overall	72.50 (25.50)	3,683	3.00 (3.00)	189	5.13 (4.45-5.91)	0.00 (1.00)	21	0.57 (0.36-0.89)	0.00 (0.00)	14	0.38 (0.21-0.65)
Pre-Intervention	69.0 (23.50)	1,852	2.00 (3.00)	68	3.67 (2.88-4.66)	0.00 (1.00)	9	0.49 (0.24-0.96)	0.00 (0.00)	4	0.22 (0.07-0.59)
Post-Intervention	76.50 (30.75)	1,831	4.50 (3.25)	121	6.61 (5.53-7.87)	0.00 (1.00)	12	0.66 (0.36-1.18)	0.00 (1.00)	10	0.55 (0.28-1.04)
**Site Two**											
Overall	53.50 (15.75)	2,618	1.50 (2.00)	123	4.70 (3.93-5.60)	0.00 (1.00)	20	0.76 (0.48-1.20)	2.00 (2.00)	94	3.59 (2.93-4.39)
Pre-Intervention	52.50 (19.25)	1,219	1.00 (2.00)	40	3.28 (2.38-4.48)	0.00 (1.00)	8	0.66 (0.31-1.34)	1.00 (2.00)	38	3.12 (2.24-4.30)
Post-Intervention	57.00 (19.50)	1,399	2.50 (3.00)	83	5.93 (4.78-7.33)	0.00 (1.00)	12	0.86 (0.47-1.54)	2.00 (3.00)	56	4.00 (3.06-5.20)
**Site Three**											
Overall	114.50 (28.00)	6,144	4.00 (4.25)	238	3.87 (3.41-4.39)	0.00 (1.00)	41	0.67 (0.49-0.91)	1.00 (3.00)	83	1.35 (1.08-1.68)
Pre-Intervention	114.00 (31.00)	3,068	2.00 (3.00)	72	2.34 (1.85-2.96)	0.00 (1.00)	8	0.26 (0.12-0.54)	0.00 (1.00)	18	0.59 (0.36-0.95)
Post-Intervention	117.50 (31.75)	3,076	5.50 (5.00)	166	5.40 (4.64-6.27)	1.00 (1.25)	33	1.07 (0.75-1.52)	2.00 (3.00)	65	2.11 (1.65-2.70)

*BMD = bone mineral density; IQR = interquartile range; CI = confidence interval.

^†^Combined across the three sites.

^‡^Rate is provided per 100 eligible visits.

### Secondary outcomes—patient-level analysis

The RAQ was completed by 351 patients (mean age 64 years; range, 50-90 years; 77% women), who represented 16% of age-eligible patients with at least one physician visit during the intervention period (Table 4). The mean time to complete the RAQ was 3.43 minutes (range, 1.32-8.01 minutes). Of those who completed the RAQ, 276 patients (79%) had at least one major risk factor for osteoporosis (*e.g.*, vertebral compression fracture, family history of maternal hip fracture) or two minor risk factors (*e.g.*, rheumatoid arthritis, smoker, weight <57 kg); in addition, 204 patients (58%) had a previous BMD test (evenly distributed between those who had the test more than two years ago and less than two years ago), and 147 patients (42%) had never had a BMD test. More men than women indicated never having a BMD test (71% vs 33%, p <0.001). Thirty-two patients (9%) reported already taking an osteoporosis medication such as a bisphosphonate (6%), hormone replacement therapy (5%), or selective estrogen reuptake modulator (0.3%); and 233 patients (66%) reported already taking calcium (8%), vitamin D (15%), or both (43%). There was a significant difference between men and women for mean age and osteoporosis risk (p <0.001 for both), but this was expected as the age-eligibility criterion for men (*i.e.*, age ≥ 65 years) was also considered a major risk factor for osteoporosis. A total of 195 patients (56%) had any disease management addressed by their physician in response to the tool at the point of care (N = 86) or within three months of the RAQ visit (N = 109). We did not analyze fractures because the incidence across sites was too low to calculate a clinically meaningful difference between the baseline and intervention periods (*i.e.*, six versus three fractures, respectively).

**Table 4:** Characteristics of patients who completed the risk assessment questionnaire (n = 351)*

**Characteristics**	**Site 1**	**Site 2**	**Site 3**	**Combined Sites**	***P*** **value** ^**†**^	
					**Between sites**	**Between women and men**
No. (%) of patients^‡^	110 (21)	124 (21)	117 (11)	351 (16)	NA	
Women	81 (74)	93 (75)	98 (84)	272 (77)	0.13	NA
Men	29 (26)	31 (25)	19 (16)	79 (23)		
Age, mean (SD), y	64 (9.56)	64 (10.40)	64 (10.55)	64 (10.2)	0.88	<0.001
Age range, y	50-87	50-90	50-89	50-90	NA	
Time to complete RAQ, mean (SD), min	3.26 (1.05)	3.42 (1.29)	3.60 (1.28)	3.43	0.08	0.37
Range of completion time, mean, min	1.32-7.93	1.37-8.01	1.37-7.38	1.32-8.01	NA	
Number of visits, mean (SD)	3.29 (1.95)	2.37 (1.85)	3.25 (3.14)	2.95 (2.4)	0.004	0.88
Range^§^	0-10	0-9	0-25	0-25	NA	
Number (%) of patients who had a BMD test						
Over 2 y ago	39 (35)	38 (31)	24 (21)	101 (29)	0.03	<0.001
Less than 2 y ago	38 (35)	30 (24)	35 (30)	103 (29)	0.22	0.005
Never	33 (30)	56 (45)	58 (50)	147 (42)	0.008	<0.001
Number (%) of patients at risk for osteoporosis^¶^	88 (80)	97 (78)	91 (78)	276 (79)	0.91	<0.001
Number (%) of patients already receiving therapy						
Osteoporosis medications	8 (7)	13 (10)	11 (9)	32 (9)	0.69	0.02
Any supplement (calcium or vitamin D)	71 (65)	81 (65)	81 (69)	233 (66)	0.72	<0.001

*All data based on Risk Assessment Questionnaire (RAQ) inputs; SD = standard deviation; BMD = bone mineral density; NA = not applicable.

^†^Based on analysis of variance or chi-square tests.

^‡^Percentage calculated as the number of age-eligible patients who completed the RAQ divided by the number of age-eligible patients with at least 1 physician visit. All percentages in subsequent rows calculated with respect to the number of age-eligible patients who completed the RAQ at the particular site.

^§^The lower limit of the range is 0 because **s**ome patients completed the RAQ during a ‘non-visit’ (*i.e.*, visited the practice but did not see a physician or nurse).

^¶^At-risk for osteoporosis is defined as 1 major or 2 minor risk factors according to clinical practice guidelines [30].

## Discussion

This study showed that a multicomponent Op-KT tool incorporating all disease management components (assessment, diagnosis and treatment) is feasible for use at the point of care. Use of the tool increased the initiation of BMD testing and treatment with medications such as bisphosphonates and vitamin D for patients at risk for osteoporosis. Our findings are consistent with other studies investigating osteoporosis disease management [38–40]. However, they also highlight continuing gaps: specifically, 42% of at-risk patients who completed the RAQ reported never having had a BMD test, and the rate of osteoporosis investigations was significantly lower among men than women (consistent with other reports [18,41]). Persistence of these gaps may be explained, in part, by how interventions are designed to overcome them. Few osteoporosis interventions include all components of disease management. Our previous systematic review found interventions with different combinations of disease management recommendations in the form of reminders and education (electronic, paper-based, or counseling-based), but none addressed all three aspects of disease management [26]. More recently, tools that do address disease management completely have been developed for high-risk [38] and postfracture [41] patients, but they don’t address how these tools might be implemented over the long term or how they might be sustainable. Furthermore, few of the existing tools use a computer-based platform for decision support [42], most are not designed to deliver evidence-based messages at the point of care, and none consider mechanisms to avoid disruption to usual practice (which can facilitate sustainability). Most tools involve coordinator-based systems (where dedicated case managers such as nurses, orthopedic surgeons, or other allied healthcare professionals are involved in facilitating care [38,39,43,44]), use telephone-based reminders [40,45], or provide reminders or education to physicians and patients through a paper-based [46,47] or counseling-based [48] strategy. These interventions can be resource intensive and therefore may not be sustainable.

Our study met the clinical goal of the osteoporosis tool, which was to increase osteoporosis investigations and treatment. Although the improvements in disease management were modest, they represent significant change in the right direction. Furthermore, the impact was relevant because the Op-KT tool was not tested under controlled conditions. It was designed and implemented as a pragmatic tool, so for example, the observed 3.2% increase in BMD testing represents a true measure of how the tool functioned in ‘real’ practice. We considered pragmatism in the tool design because our foundational work indicated that such tools might unintentionally disrupt the real reason for the visit under more controlled conditions (*i.e.*, if a prompting mechanism was enforced for every at-risk patient) [28]. Healthcare professionals prefer the provision of guidance that does not interfere with usual care [49], as confirmed by our investigation of practice workflows across the three sites, so we deliberately designed the tool to *not* prompt patients to complete the risk questionnaire. This also meant that the tool was not initiated during every relevant ‘at-risk’ encounter, which resulted in a relatively low rate of use (16% across the three sites). However, physicians addressed the disease management of more than half of these patients, which indicates that the tool has great potential to enhance care. The challenge in designing KT tools such as this one is to find the right balance between transmitting appropriate practice knowledge at the right time and ensuring that more urgent aspects of the visit agenda (*e.g.*, chest pain) or health status (*e.g.*, diabetes complication) are not disrupted, particularly in practice settings where visits are short and involve multiple complaints requiring complex clinical decisions.

Our positive findings also highlight several unique features that can be considered when designing clinical decision support systems such as the Op-KT tool. First, the information that our tool delivered to physicians and patients was customized according to the risk profile of each individual patient. The tool functions by extracting and analyzing risk inputs according to a guideline-based disease management algorithm; and then deliver relevant, evidence-based prompts to aid physicians in their clinical decision making (BestPROMPT), and provide a risk profile for patients with self-management options tailored to their own risks (COPE). Very few existing tools have all of these features. FRAX, a widely known risk assessment tool, is designed to estimate individualized 10-year probability of hip and major osteoporotic fractures [50], and has been calibrated for populations in Canada [51] and the United States [52]. It estimates the 10-year fracture risk by integrating age, gender, and seven clinical risk factors independent of BMD [50], but it was not designed to consider the treatment recommendations that would be relevant to the risks identified, nor can it suggest tailored messages for patients to facilitate self-management. Additionally, evidence from many studies has shown that guidelines on their own are not sufficient to change practice [53] and decision support tools, such as the one we created, provide potential solutions to this challenge.

Second, our finding that patient involvement in disease management is feasible at the point of care highlights the untapped opportunities to improve care in primary care settings. A recent systematic review of quality improvement strategies for diabetes mellitus found that interventions targeting the disease management system along with patient-mediated strategies improved care [54]. Another recent analysis showed that clinical decision support systems that provide advice for patients in addition to practitioners are more likely to succeed [55]. Lack of time is a major barrier to implementing clinical decision support tools in family practices [56,57], so it’s prudent to shift some of the disease management tasks to patients. In our study, we showed that this is possible, as patients were able to complete the RAQ in the waiting room or in the examination room prior to their visit. Involving patients in the continuum of care can facilitate self-management, making it easier for patients to receive important information and to gain the skills and confidence (self-efficacy) to deal with their illness [58]. Moreover, patient self-management may facilitate the sustainability of an intervention by alleviating resource burdens that might be needed to maintain continuing use of the tool. Indeed, elderly patients are the fastest-growing population group [59], a demographic shift that is expected to increase the prevalence of chronic diseases [60] and the need for patient self-care to support disease management [60,61].

This study also made several contributions to the development of complex interventions for chronic disease where the goal is to effectively translate best evidence at the point of care. The tool underwent substantial usability testing before implementation [28]. Moreover, workflow analysis preceded its roll-out in the family practice units to optimize use of the tool. These efforts were perceived to have been successful, because the patients who initiated the RAQ were able to complete it rapidly (mean 3.43 minutes). Lastly, the Op-KT tool was developed from a robust evidentiary base informed by two theoretical frameworks (the knowledge-to-action and MRC frameworks). These not only helped in mapping the process for translating osteoporosis evidence into practice, they also facilitated the selection of appropriate study designs to answer our questions, to anticipate barriers, to evaluate outcomes, and to address implementation and sustainability barriers [62].

Our study had some limitations. This was a pilot study, so our sample sizes were smaller than would be expected if we had conducted a more rigorous randomized controlled trial (RCT). As a result, findings may not be generalizable to other family practice settings or populations. However, in ITS studies, sample size calculations are related to the estimation of the number of observations or time points at which data are collected rather than the number of sites, physicians, and patients. As such, we included a higher number of data points (*i.e.*, 26 data points before and 26 data points after the introduction of the intervention) than recommended (20 data points before/after) to ensure enough power to detect a change and to account for threats to internal validity [32,33]. Furthermore, data points were set closer together (*i.e.*, each data point represented a two-week period rather than four weeks) to increase validity [34]. We recognize that the ITS methodology is inherently more susceptible to validity threats than would be a more rigorous RCT design. However, for a pilot evaluation, this methodology was most appropriate, and we designed our study according to rigorous ITS criteria [32,33] to help rule out alternative explanations of our findings. Third, the guideline used in the study was an earlier version [30], so the recommendations are not consistent with current guidelines. However, the tool is currently being updated to reflect these changes. Although we planned to integrate our tool within the PracticeSolutions system, this was not possible because of programming and EMR proprietary barriers. Such integration can facilitate more efficient delivery of patient-specific decision support at the point of care [38,57,63], and a more automated transfer of data while preserving the accuracy of patient-reported risks. However, we showed that a stand-alone, partially electronic system could benefit clinical practice. In addition, the majority of current practices are paper-based, so our tool is more generalizable to these settings now, but has great potential to be transformed into a fully electronic tool as physician practices become increasingly paperless.

## Conclusions

Our multicomponent Op-KT tool significantly increased osteoporosis disease management in three family practices. This study highlights the potential of using decision support tools at the point of care in busy, short-visit practices to facilitate patient self-management.

## Additional files

Additional file 1:
**Screenshots of the Risk Assessment Questionnaire (RAQ).**
Additional file 2:
**Screen shots of the Best Practice Recommendation Prompt (BestPROMPT).**
Additional file 3:
**Screen shot of the Customized Osteoporosis Education (COPE) sheet.**
