# Supplementary material for: A knowledge translation tool improved osteoporosis disease management in primary care: an interrupted time series analysis
Source: Implement Sci. 2014 Sep 25;9:109. doi: 10.1186/s13012-014-0109-9 (PMC4182792; doi:10.1186/s13012-014-0109-9)
Supplement: Additional file 1: — Screenshots of the Risk Assessment Questionnaire (RAQ). [file 13012_2014_109_MOESM1_ESM.doc]

**Appendix 1**

Screenshots of the Risk Assessment Questionnaire (RAQ)

The functional Op-KT tool is also accessible at: <http://knowledgetranslation.ca/osteo_final>

| **RAQ component** | **Screen shot** |
| --- | --- |
| **Screen shot of RAQ touch screen tablet device that was used in the study** | 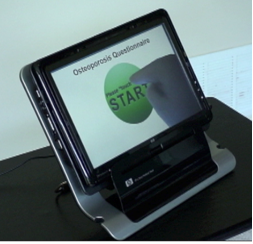 |
| **Screen shot of RAQ interface:** *Participant age* | 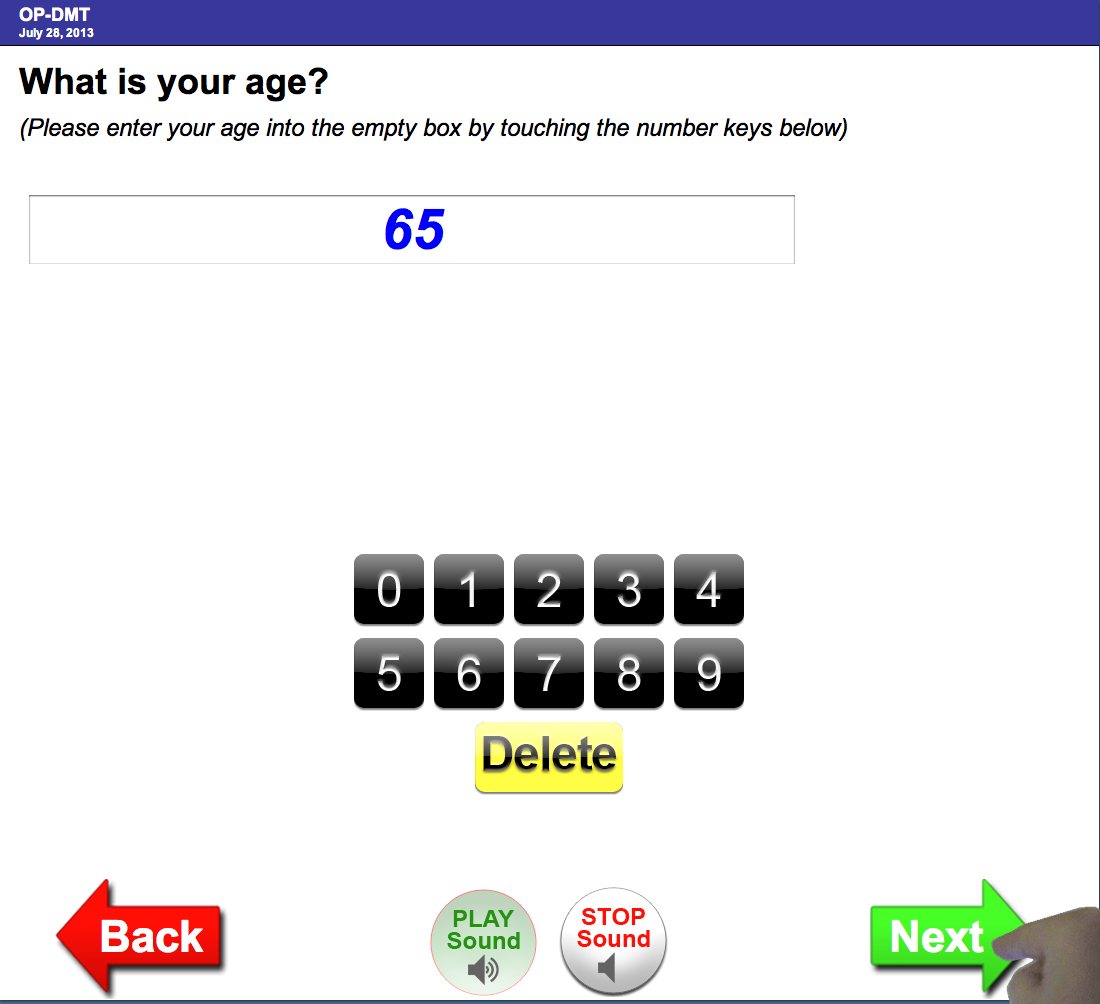 |
| **Screen shot of RAQ interface:** *Falls risk assessment* | 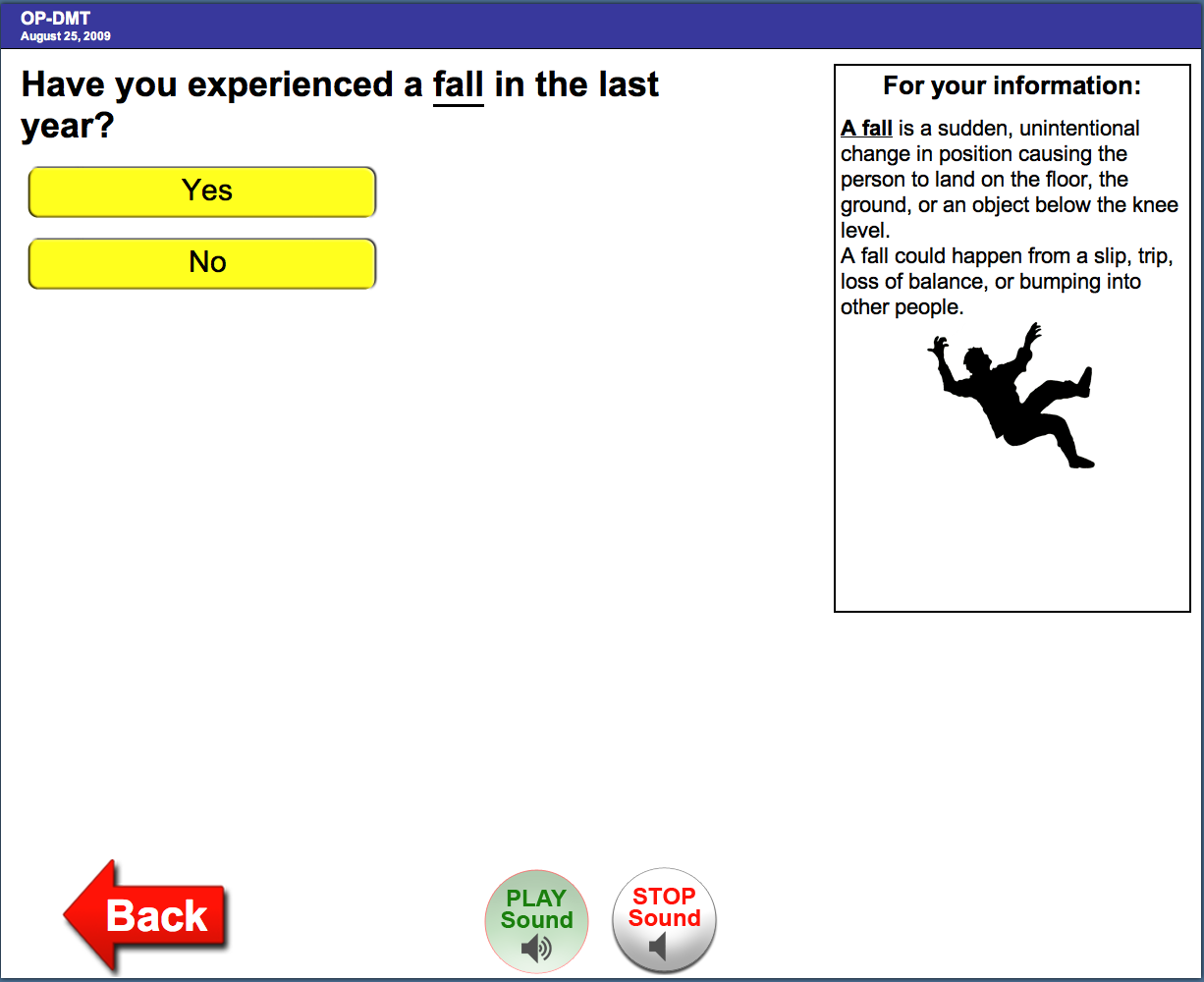 |
| **Screen shot of RAQ interface:** *Assessment for at-risk conditions* | 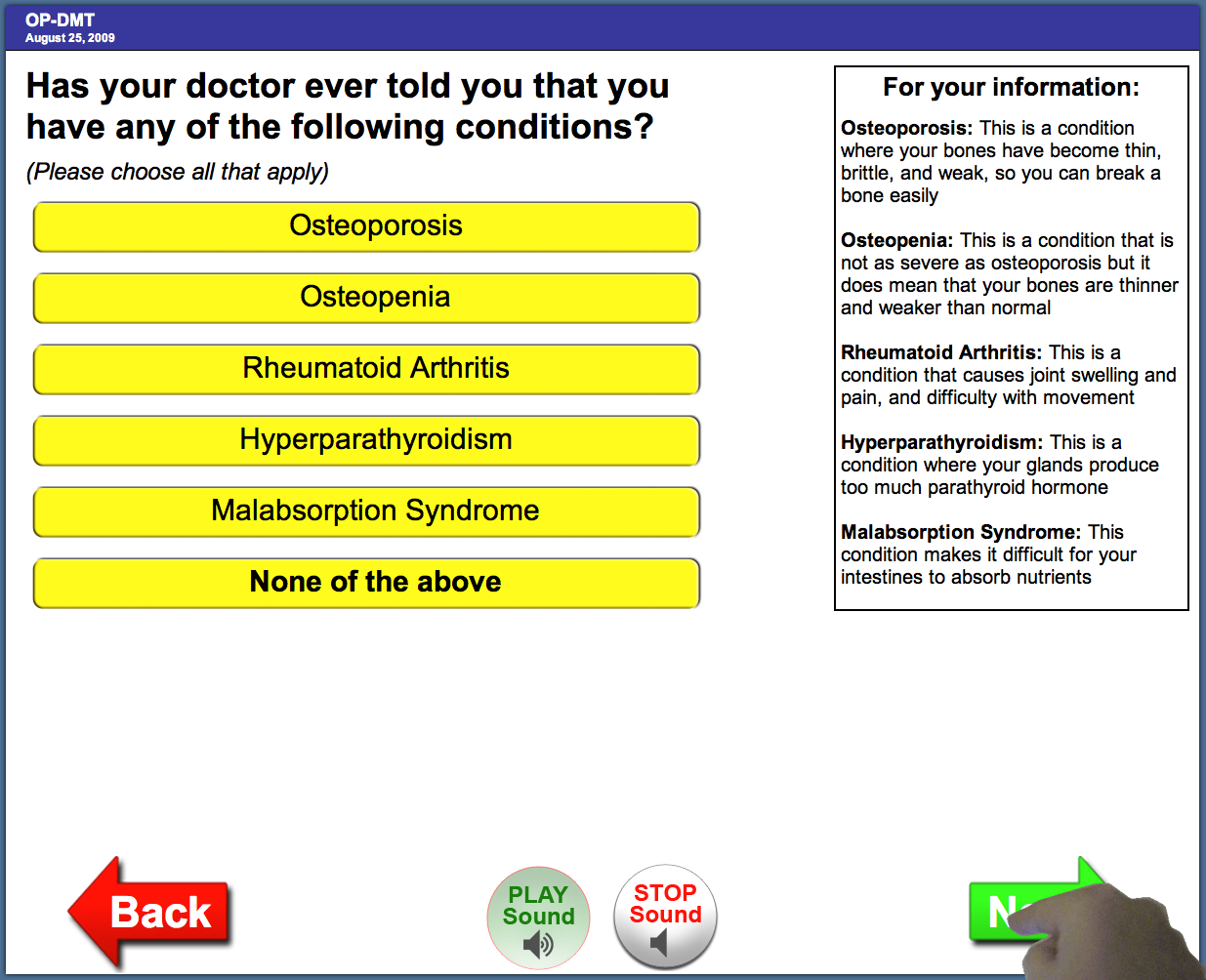 |
