# Supplementary material for: A knowledge translation tool improved osteoporosis disease management in primary care: an interrupted time series analysis
Source: Implement Sci. 2014 Sep 25;9:109. doi: 10.1186/s13012-014-0109-9 (PMC4182792; doi:10.1186/s13012-014-0109-9)

**Appendix 2**

Screen shots of the Best Practice Recommendation Prompt (BestPROMPT)

The functional Op-KT tool is also accessible at: <http://knowledgetranslation.ca/osteo_final>


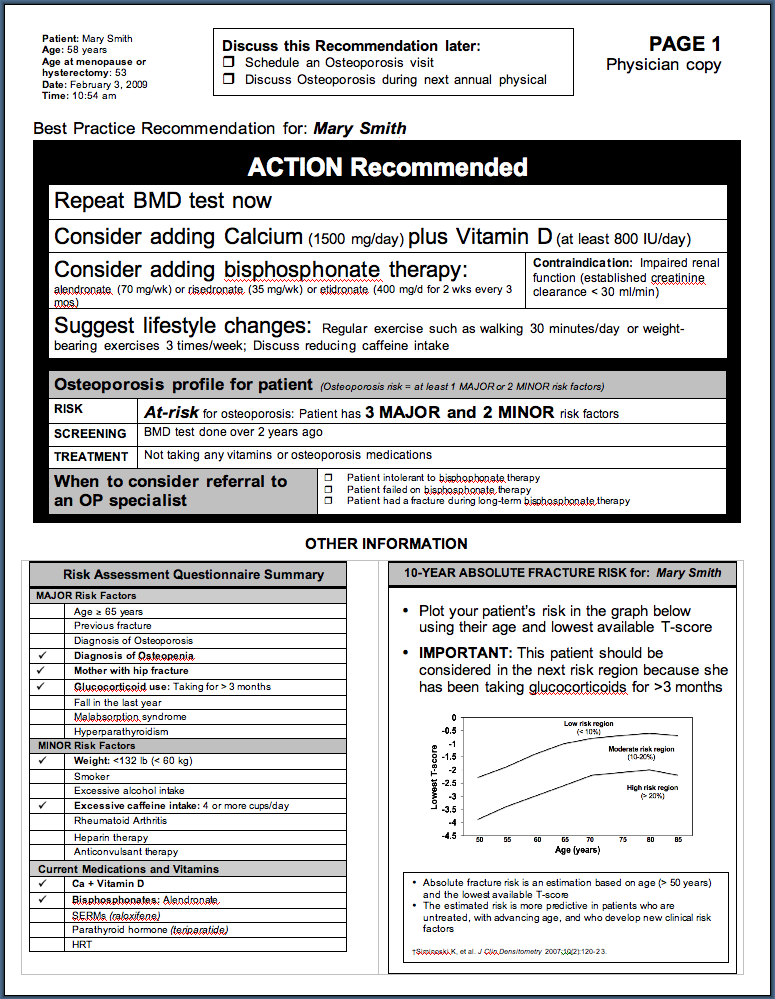

Supplement: Additional file 2: — Screen shots of the Best Practice Recommendation Prompt (BestPROMPT). [file 13012_2014_109_MOESM2_ESM.doc]
