# Supplementary material for: A knowledge translation tool improved osteoporosis disease management in primary care: an interrupted time series analysis
Source: Implement Sci. 2014 Sep 25;9:109. doi: 10.1186/s13012-014-0109-9 (PMC4182792; doi:10.1186/s13012-014-0109-9)

**Appendix 3**

Screen shot of the Customized Osteoporosis Education (COPE) sheet

The functional Op-KT tool is also accessible at: <http://knowledgetranslation.ca/osteo_final>


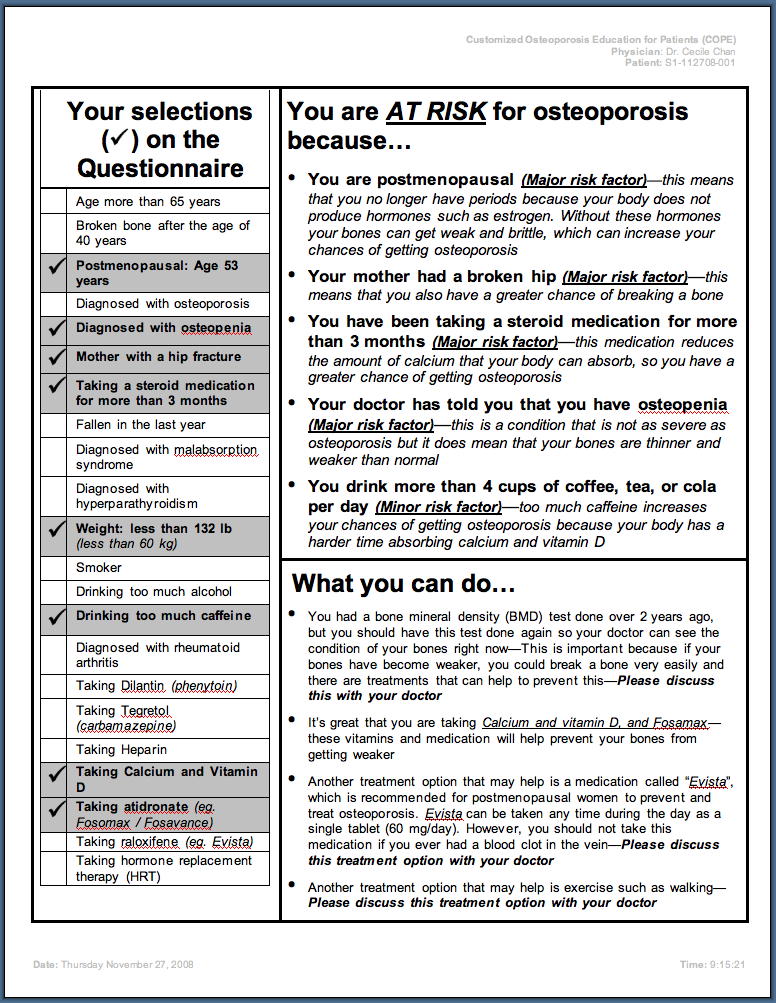

Supplement: Additional file 3: — Screen shot of the Customized Osteoporosis Education (COPE) sheet. [file 13012_2014_109_MOESM3_ESM.doc]
